# Incorporating Baseline Outcome Data in Individual Participant Data Meta-Analysis of Non-randomized Studies

**DOI:** 10.3389/fpsyt.2022.774251

**Published:** 2022-02-22

**Authors:** Lamprini Syrogiannouli, Lea Wildisen, Christiaan Meuwese, Douglas C. Bauer, Anne R. Cappola, Jacobijn Gussekloo, Wendy P. J. den Elzen, Stella Trompet, Rudi G. J. Westendorp, J. Wouter Jukema, Luigi Ferrucci, Graziano Ceresini, Bjørn O. Åsvold, Layal Chaker, Robin P. Peeters, Misa Imaizumi, Waka Ohishi, Bert Vaes, Henry Völzke, Josè A. Sgarbi, John P. Walsh, Robin P. F. Dullaart, Stephan J. L. Bakker, Massimo Iacoviello, Nicolas Rodondi, Cinzia Del Giovane

**Affiliations:** ^1^Institute of Primary Health Care (BIHAM), University of Bern, Bern, Switzerland; ^2^Department of Intensive Care Medicine, University Medical Centre Utrecht, Utrecht, Netherlands; ^3^Departments of Medicine and Epidemiology and Biostatistics, University of California, San Francisco, San Francisco, CA, United States; ^4^Division of Endocrinology, Diabetes, and Metabolism, Department of Medicine, University of Pennsylvania School of Medicine, Philadelphia, PA, United States; ^5^Section of Gerontology and Geriatrics, Department of Internal Medicine, Leiden University Medical Center, Leiden, Netherlands; ^6^Department of Public Health and Primary Care, Leiden University Medical Center, Leiden, Netherlands; ^7^Atalmedial Diagnostics Centre, Amsterdam, Netherlands; ^8^Department of Clinical Chemistry, Amsterdam Public Health Research Institute, Amsterdam UMC, Amsterdam, Netherlands; ^9^Department of Public Health and Center for Healthy Aging, University of Copenhagen, Copenhagen, Denmark; ^10^Department of Cardiology, Leiden University Medical Center, Leiden, Netherlands; ^11^Netherlands Heart Institute, Utrecht, Netherlands; ^12^Longitudinal Studies Section, Translational Gerontology Branch, National Institute on Aging, Baltimore, MD, United States; ^13^Unit of Internal Medicine and Onco-Endocrinology, Department of Medicine and Surgery, University Hospital of Parma, Parma, Italy; ^14^Department of Public Health and Nursing, K.G. Jebsen Center for Genetic Epidemiology, NTNU, Norwegian University of Science and Technology, Trondheim, Norway; ^15^Department of Endocrinology, Clinic of Medicine, St. Olavs Hospital, Trondheim University Hospital, Trondheim, Norway; ^16^Department of Internal Medicine, Erasmus University Medical Center, Rotterdam, Netherlands; ^17^Department of Epidemiology, Erasmus University Medical Center, Rotterdam, Netherlands; ^18^Academic Center for Thyroid Diseases, Erasmus University Medical Center, Rotterdam, Netherlands; ^19^Department of Clinical Studies, Radiation Effects Research Foundation, Nagasaki, Japan; ^20^Department of Clinical Studies, Radiation Effects Research Foundation, Hiroshima, Japan; ^21^Department of Public Health and Primary Care, KU Leuven, Leuven, Belgium; ^22^Institute for Community Medicine, Clinical-Epidemiological Research, University Medicine Greifswald, Greifswald, Germany; ^23^Division of Endocrinology and Metabolism, Department of Medicine, Faculdade de Medicina de Marilia, São Paulo, Brazil; ^24^Medical School, The University of Western Australia, Crawley, WA, Australia; ^25^Department of Endocrinology and Diabetes, Sir Charles Gairdner Hospital, Nedlands, WA, Australia; ^26^Department of Internal Medicine, University Medical Center, University of Groningen, Groningen, Netherlands; ^27^Cardiology Unit, University Hospital Policlinico Consorziale of Bari, Bari, Italy; ^28^Department of General Internal Medicine, Inselspital, Bern University Hospital, University of Bern, Bern, Switzerland; ^29^Population Health Laboratory (#PopHealthLab), University of Fribourg, Fribourg, Switzerland

**Keywords:** individual participant data, continuous outcome, non-randomized studies, cohorts, baseline imbalance

## Abstract

**Background:**

In non-randomized studies (NRSs) where a continuous outcome variable (e.g., depressive symptoms) is assessed at baseline and follow-up, it is common to observe imbalance of the baseline values between the treatment/exposure group and control group. This may bias the study and consequently a meta-analysis (MA) estimate. These estimates may differ across statistical methods used to deal with this issue. Analysis of individual participant data (IPD) allows standardization of methods across studies. We aimed to identify methods used in published IPD-MAs of NRSs for continuous outcomes, and to compare different methods to account for baseline values of outcome variables in IPD-MA of NRSs using two empirical examples from the Thyroid Studies Collaboration (TSC).

**Methods:**

For the first aim we systematically searched in MEDLINE, EMBASE, and Cochrane from inception to February 2021 to identify published IPD-MAs of NRSs that adjusted for baseline outcome measures in the analysis of continuous outcomes. For the second aim, we applied analysis of covariance (ANCOVA), change score, propensity score and the naïve approach (ignores the baseline outcome data) in IPD-MA from NRSs on the association between subclinical hyperthyroidism and depressive symptoms and renal function. We estimated the study and meta-analytic mean difference (MD) and relative standard error (SE). We used both fixed- and random-effects MA.

**Results:**

Ten of 18 (56%) of the included studies used the change score method, seven (39%) studies used ANCOVA and one the propensity score (5%). The study estimates were similar across the methods in studies in which groups were balanced at baseline with regard to outcome variables but differed in studies with baseline imbalance. In our empirical examples, ANCOVA and change score showed study results on the same direction, not the propensity score. In our applications, ANCOVA provided more precise estimates, both at study and meta-analytical level, in comparison to other methods. Heterogeneity was higher when change score was used as outcome, moderate for ANCOVA and null with the propensity score.

**Conclusion:**

ANCOVA provided the most precise estimates at both study and meta-analytic level and thus seems preferable in the meta-analysis of IPD from non-randomized studies. For the studies that were well-balanced between groups, change score, and ANCOVA performed similarly.

## Introduction

In non-randomized studies (NRS) that assess a continuous outcome of interest (e.g., depressive symptoms) at baseline and follow-up, baseline values between treatment or exposure and control group may differ significantly. Ignoring this imbalance in the analysis may confound the estimated study effect (1). Likewise, when there is correlation between baseline values and change score (the difference between follow-up and baseline values), the researchers performing the statistical analysis must take this into account. Failing to do so may reduce the precision and increase risk of bias in study results (1). For example, in a study that assesses the effect of a treatment compared to a control using a continuous outcome over a certain period of follow-up, we may observe that people in the treatment group have higher baseline value of the outcome variable than those in the control group. Furthermore, we may also see that baseline outcome values (e.g., depressive symptoms measured at baseline) correlate positively with the difference between follow-up and baseline (higher baseline values change more in absolute terms, i.e., regression to the mean). In this case, the treatment (e.g., antidepressant medication) will appear more effective than it truly is (2, 3). This problem can be avoided by accounting for baseline imbalances between the groups and for this type of correlation when we analyze continuous outcomes in NRSs. A few statistical methods are available to deal with this issue.

The most common methods are analysis of covariance (ANCOVA) and the change score. These methods are both based on a linear regression model (1, 4). ANCOVA uses follow-up values as outcome, adjusted for baseline values. In the change score, baseline outcome values are included in the outcome definition of the model: the outcome is the difference between follow-up and baseline values. There has been extensive debate over which approach is preferable and the question is still controversial (2, 4–6).

Another method that may account for baseline imbalance in study analysis is the propensity score, also called inverse probability weighing, which accounts for baseline imbalances by assigning weights to each participant. In this method, the researcher applies a linear regression model with follow-up values as outcome and each participant is weighted for the conditional probability of being treated or exposed, given the baseline outcome. Weights are calculated as the inverse probability of being treated/exposed given baseline outcome values, under the assumption of no unmeasured confounders that may affect the estimate and the causal effect of the exposure (7, 8). This method weights participants who were unlikely to receive the treatment (or being exposed) higher than those who were likely to receive the treatment but did not.

Another issue is that the pooled estimate obtained from the MA of NRSs with biased estimates due to ignoring imbalance at baseline in the study statistical analysis may also be biased, as well as less efficient (9, 10). If studies included in the MA used different methods (e.g., ANCOVA, change score, or propensity score) to analyze continuous outcomes, the pool result could be influenced by aggregate estimates that were derived differently. This problem can be solved by standardizing the analytic approach across studies included in the MA using individual participant data (IPD) instead of aggregate study data (9, 10). MA of IPD is increasingly common and is now considered the best method for combining study results (11). Riley et al. (1) compared studies and meta-analytic estimates between ANCOVA and change score in IPD MA of RCTs by assuming different scenarios of baseline imbalance between groups. We found no research that measured the effects of the propensity score method at the study and meta-analytic level by comparing ANCOVA to change score and none that compared the effect of ANCOVA and change score in study and meta-analytic estimates from IPD-MA of NRSs.

Our first aim was to identify the statistical methods IPD MA of NRSs used to deal with continuous outcomes assessed at baseline and follow-up. Our second aim was to compare the impact of the above methods in the study and meta-analytic estimates in two empirical examples of IPD-MA of NRSs.

## Methods

To identify the various statistical methods, we systematically reviewed published IPD-MAs of NRSs that analyzed continuous outcomes and used baseline outcome data in the analysis. We built the search strategy with the help of a medical librarian. We searched Medline (PubMed), Embase (Ovid), and CENTRAL (Cochrane Library) from inception to February 2021 using the key terms listed in the Supplementary Materials. In addition to completed studies, study protocols of IPD-MAs of NRSs were eligible for inclusion. We excluded methodological studies like those that assessed the effect of different statistical methods on the results of IPD-MA of NRSs that incorporated baseline outcome data in analysis of continuous outcomes. We placed no restrictions on study population or underlying medical conditions. We imported search results into a citation manager (https://rayyan.qcri.org/) and removed duplicates. Two authors (LS and LW) independently screened citations by title and abstract against predefined eligibility criteria. The same two authors reviewed the full text of all selected records. They resolved disagreements by discussion and, if needed, consulted a third author (CDG) to reach consensus. From each eligible IPD-MA, we extracted the following information: number of included cohorts/studies; number of participants; clinical field; assessment of potential outcome baseline imbalance between groups; assessment of the correlation between baseline and follow-up outcome data; primary statistical method that accounted for baseline outcome data, and eventual method used in a secondary analysis. We piloted an electronic data extraction form that was used by the two reviewers to extract information of interest from included publications.

For our second aim we used data from the Thyroid Studies Collaboration (TSC): (1) Wildisen et al. assessed the association between subclinical hyperthyroidism (exposure) and depressive symptoms (outcome) (12), and (2) Meuwese et al. on the association between overt and subclinical hyperthyroidism (exposure) and renal function (outcome) (13). Each study included in each publication was approved by its local ethics committee and all participants gave informed consent for the original studies. Participants with subclinical hyperthyroidism were defined as those with thyroid stimulating hormone (TSH) <0.45 mIU/L and normal free thyroxine (FT4) (14). For both examples, we considered euthyroid participants (TSH levels between 0.45 and 4.49 mIU/L and normal FT4 levels; reference range from original studies) as members of the unexposed group.

We included cohorts with available data on the outcome of interest (depressive symptoms or renal function) at baseline, at first available follow-up, and with thyroid status at baseline (measured TSH). Depressive symptoms were measured on a validated depression scale in the Beck Depression Inventory (BDI). BDI scales go from 0 to 63; higher values indicate more symptoms of depressive symptoms (15). We measured renal function with estimated glomerular filtration rates (eGFR) in mL/min/1.73m^2^; values lower than 60 mL/min/1.73m^2^ indicate deteriorated renal function. eGFR was calculated with the four-variable Modification of Diet in Renal Disease formula when it was not in the original source data.

We analyzed only participants whose baseline and follow-up data were both available. We also collected data on age and sex for each cohort. We calculated the mean and standard deviation (SD) of the continuous outcomes at baseline and follow-up in each cohort study and assessed statistical baseline imbalances between groups with the *t*-test. We verified the data were normally distributed. For each cohort, we also calculated the correlation coefficient between baseline and follow-up outcome data. Then we executed a two-stage IPD-MA. In the first stage, we estimated the study-specific mean difference (MD) of the outcome between participants with subclinical hyperthyroidism and euthyroid participants and, to measure the precision of the estimates, the relative standard error (SE). We obtained study estimates from ANCOVA, change score, and propensity score. For comparison, we also applied the naïve approach, which model follow-up outcome data and ignores baseline outcome data. Naïve model has been showed to produce biased estimates in case of the presence of baseline imbalance (1). Since we used NRSs and therefore other baseline variables may have operated as confounders we additionally adjusted for age and sex in each method to have more reliable results. The statistical model for each method, without adjustment for age and sex for each method, is presented in the Supplementary Materials. Finally, we pooled the MDs across studies using both fixed and random effects meta-analysis to derive the meta-analytic estimates reported again as MD, SE, and relative 95% confidence interval (CI). Between-study variance was estimated by τ^2^; we also calculated the *I*^2^ as measure of heterogeneity. All analyses were performed in STATA v15 (StataCorp. 2017. Stata Statistical Software: Release 15. College Station, TX: StataCorp LLC.).

## Results

### Systematic Review of IPD-MAs of NRSs

**Figure 1:**
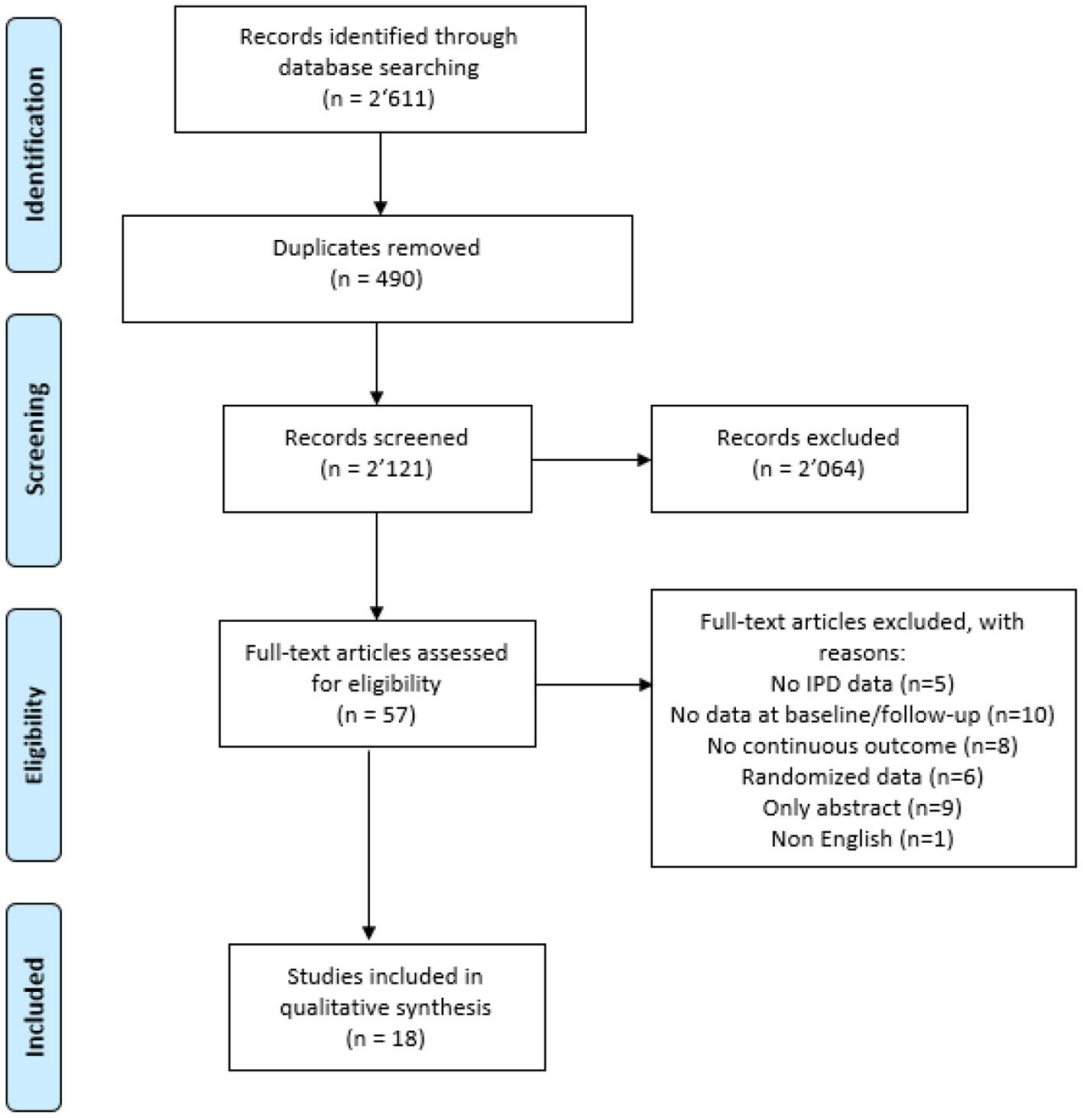
Flow chart.

Our initial search yielded 2,611 unique citations, which we scrutinized for eligibility. Figure 1 contains the flow chart of study identification. We included 18 publications of IPD-MA of NRSs evaluating continuous outcomes (12, 13, 16–31), more than half (61%) published since 2018. Table 1 lists the characteristics of the studies we included: 10 (56%) used change score; seven (39%) used ANCOVA, and one (5%) used propensity score. No study assessed the presence of baseline outcome imbalance between groups or correlation between baseline and follow-up data.

**Table 1:** Characteristics of included individual participant data meta-analysis of non-randomized studies.

**References**	**Clinical field**	**Number of studies/patients**	**Outcome**	**Assessment of baseline imbalance between groups**	**Assessment of the correlation between baseline and follow-up**	**Method used to account for baseline outcome values**	**Other methods used as sensitivity analysis**
Kelley and Kelley (24)	Endocrinology	3/143	Bone mineral density values	No	No	ANCOVA	No
Holloway et al. (23)	Neurology	24/137	Burke-Fahn-Marsden movement scale for deep brain stimulation	No	No	ANCOVA	No
Chambrone et al. (21)	Periodontology	1/52	Probing depths	No	No	ANCOVA	Naive
Mosges et al. (22)	Allergiology	10/140,853	Four antihistamines alone or in combination with intranasal corticosteroids	No	No	Change score	No
Willeit et al. (20)	Cardiology	20/49,097	common-carotid-artery intima-media thickness	No	No	Change score	No
Zaghi et al. (19)	Surgery	45/518	Apnea-hypopnea index and respiratory disturbance index	No	No	Change score	No
Stafford et al. (18)	Mental Health	4/7,515	Positive mental wellbeing	No	No	ANCOVA	No
Segna et al. (17)	Internal Medicine/ Endocrinology	6/5,458	Bone mineral density change	No	No	Change score	No
Elkaim et al. (16)	Neurology	72/321	Burke-Fahn-Marsden or Barry-Albright Dystonia Scale Scores	No	No	Change score	No
Westerhausen and Karud (31)	Neurology	16/87	Intelligence test performance	No	No	Change score	No
Driessen et al. (30)^*^	Psychology	–	Depressive symptoms	–	–	ANCOVA	No
Meuwese et al. (13)	Nephrology/ Endocrinology	16/72,856	Glomerular filtration rates	No	No	Change score	No
Coulombe et al. (29)	Neurology	21/58	Yale Global Tic Severity Scale score	No	No	Change score	No
Kuramatsu et al. (27)	Surgery	4/578	Cerebellar Intracerebral Hemorrhage functional disability	No	No	Propensity score	ANCOVA
Poole et al. (28)	Neurology	7/766	Intracranial pressure (ICP)	No	No	ANCOVA	No
Wade et al. (26)	Physical activity	13/23,731	Exercise referral schemes scores	No	No	Change score	No
Wildisen et al. (12)^*^	Psychology	–	Depressive symptoms	–	–	ANCOVA	No
Palapar et al. (25)	Internal Medicine	5/2,392	Functional ability, cognitive function, depressive symptoms, and self-rated health	No	No	Change score	No

* *Indicates protocols of studies*.

*ANCOVA, Analysis of covariance*.

### Comparison of Methods on Study and Meta-Analytic Estimates From Two IPD-MA of NRSs

#### Association Between Subclinical Hyperthyroidism and Depressive Symptoms

Six studies were included in our analysis with total sample size ranging between 257 and 15,576 participants (Table 2). No studies had statistically significant outcome baseline imbalance between groups. The correlation ranges between 0.44 and 0.73 (Table 2). Results at study level for each statistical method are reported in Table 2 and those at meta-analytic level in Table 3. At study level, the study that presented largest difference between groups at baseline although not statistically significant [i.e., PROSPER (33)] had wide variation in the estimates throughout the four methods, with MD ranging from −2.06 in the naïve approach to 1.02 for the change approach (higher positive values indicate more depressive symptoms) (Table 2). The study estimates were similar across the methods in case of balanced baseline outcome data between groups [see for example Leiden 85-plus Study (32)]. For each study ANCOVA and change approach showed MDs in the same direction (e.g., positive) (Table 2). The study SEs of the ANCOVA were smaller compared to the other approaches, indicating more precise estimates, while propensity score provided the least precise study estimates (Table 2). At meta-analytic level, ANCOVA provided more precise pooled estimates in the fixed effects model (SE = 0.27) while the least precise method was the naïve approach (SE = 0.32), even though no method identified an association between depressive symptoms and subclinical hyperthyroidism (Table 3). The pooled estimates were mainly driven by HUNT (37), which is the biggest study (with very similar baseline outcome data between groups) and thus with the largest weight in the meta-analysis (% weight for HUNT >54%) (Supplementary Figure 1). In the random effect model, the propensity score approach showed more precise pooled estimate (SE = 0.31), following by the naïve approach (SE = 0.32) and ANCOVA (SE = 0.34), while the change approach had the least precise pooled estimate (SE = 0.49). Heterogeneity was highest when change score was used as outcome (τ^2^ = 0.56) compared to that from ANCOVA (τ^2^ = 0.13) and null for the propensity score and naïve approach (Table 3). In both fixed and random effects, pooled results from propensity score were more in favor to the exposure group compared to the other methods (subclinical hyperthyroidism reduced depressive symptoms in the BDI scale of 0.32 compared to the control group) (Table 3 and Supplementary Figure 2).

**Table 2:** Summary of studies that assessed the association between subclinical hyperthyroidism and depressive symptoms.

**Study**	**Number of patients**		**Depressive symptoms baseline** ***mean (SD*****)^§^**			**Depressive symptoms follow-up** ***mean (SD)***		**Correlation between baseline and follow-up**		**Naïve**	**ANCOVA**	**Change score**	**Propensity score**
	***Euthy-roid***	***Shyper***	***Euthy-roid***	***Shyper***	***p-value***	***Euthy-roid***	***Shyper***	***Euthy-roid***	***Shyper***	***MD (SE)***	***MD (SE)***	***MD (SE)***	***MD (SE)***
Leiden 85-plus Study (32)	239	18	9.65 (10.02)	9.57 (10.66)	0.97	9.82 (10.80)	13.3 (14.85)	0.70	0.73	3.44 (2.72)	3.55 (1.94)	3.58 (2.01)	3.42 (3.34)
PROSPER (33)	348	17	10.10 (7.90)	6.92 (5.18)	0.10	9.88 (8.40)	7.66 (6.38)	0.70	0.63	−2.06 (2.08)	0.22 (1.51)	1.02 (1.58)	−0.62 (1.93)
HABC (34)	2,150	81	4.77 (5.40)	5.37 (5.72)	0.33	6.72 (6.62)	7.57 (7.04)	0.47	0.44	0.51 (0.75)	0.27 (0.66)	0.09 (0.71)	0.02 (0.73)
CHS (35)	3,047	112	11.15 (10.21)	11.94 (9.69)	0.42	11.00 (10.30)	10.66 (8.12)	0.62	0.53	−0.80 (0.98)	−1.00 (0.77)	−1.13 (0.86)	−1.01 (0.75)
InChianti (36)	903	61	12.34 (8.74)	11.24 (6.58)	0.34	15.05 (8.91)	15.78 (9.08)	0.55	0.58	−0.05 (1.08)	0.72 (0.95)	1.51 (1.09)	0.44 (1.18)
HUNT (37)	15,157	419	10.77 (8.95)	11.57 (9.50)	0.07	10.93 (8.74)	11.22 (9.00)	0.55	0.54	0.18 (0.43)	−0.22 (0.36)	−0.58 (0.42)	−0.36 (0.42)

§ *The p-values from the t-test were >0.05 for all studies*.

*Shyper, subclinical hyperthyroidism; ANCOVA, analysis of covariance; MD, mean difference; SD, standard deviation; SE, standard error*.

**Table 3:** Meta-analytic results by statistical method and empirical example.

	**Depressive symptoms**	**Renal function**
**Naïve**		
**Fixed**		
*MD (SE)*	0.11 (0.32)	0.92 (0.55)
*95% CI*	(−0.53, 0.74)	(−0.16, 2.00)
**Random**		
*MD (SE)*	0.11 (0.32)	0.92 (0.55)
*95% CI*	(−0.53, 0.74)	(−0.16, 2.00)
τ^2^, *I*^2^	0.00, 0%	0.00, 0%
**ANCOVA**		
**Fixed**		
*MD (SE)*	−0.07 (0.27)	−0.20 (0.51)
*95% CI*	(−0.60, 0.47)	(−0.89, 0.49)
**Random**		
*MD (SE)*	0.00 (0.32)	−0.48 (0.53)
*95% CI*	(−0.67, 0.67)	(−1.53, 0.56)
τ^2^, *I*^2^	0.13, 18.1%	1.00, 33.3%
**Change score**		
**Fixed**		
*MD (SE)*	−0.20 (0.32)	−0.66 (0.74)
*95% CI*	(−0.80, 0.40)	(−1.38, 0.07)
**Random**		
*MD (SE)*	0.10 (0.32)	−1.51 (0.74)
*95% CI*	(−0.86, 1.05)	(−2.97, −0.05)
τ^2^, *I*^2^	0.56, 43.1%	3.18, 58.5%
**Propensity score**		
**Fixed**		
*MD (SE)*	−0.32 (0.31)	1.48 (0.56)
*95% CI*	(−0.93, 0.29)	(0.36, 2.56)
**Random**		
*MD (SE)*	−0.32 (0.31)	1.44 (0.58)
*95% CI*	(−0.93, 0.29)	(0.30, 2.58)
τ^2^, *I*^2^	0.00, 0%	0.16, 3.5%

*MD, mean difference; SD, standard deviation; SE, standard error; CI, confidence intervals*.

#### Association Between Subclinical Hyperthyroidism and Renal Function

We included 13 studies in our analysis; sample size ranged between 230 and 14.187 participants (Table 4). The *t*-test revealed statistically outcome baseline imbalance between groups in SHIP (44), PROSPER (33), InChianti (36), and HUNT (37). We also found some baseline imbalance in other studies like Bari (38), Health ABC (34), and PREVEND (41). This imbalance was not statistically significant according to the *t*-test, likely because sample size was small. We found similar baseline outcome data between groups for Belfrail (39) and Busselton (40).

**Table 4:** Individual non-randomized studies included in renal function application of the IPD MA.

**Study**	**Number of patients**		**eGFR baseline** ***mean (SD)***			**eGFR follow-up** ***mean (SD)***		**Correlation between baseline and follow-up**		**Naïve**	**ANCOVA**	**Change score**	**Propensity score**
	***Euth-yroid***	***Shyper***	***Euthy-roid***	***Shyper***	***p-value***	***Euthy-roid***	***Shyper***	***Euthy-roid***	***Shyper***	***MD (SE)***	***MD (SE)***	***MD (SE)***	***MD (SE)***
Bari (38)	221	9	74.84 (25.90)	78.12 (33.93)	0.71	73.55 (26.72)	73.70 (35.40)	0.79	0.92	3.77 (7.91)	−0.72 (5.24)	−2.38 (5.66)	−2.86 (11.81)
BELFRAIL (39)	366	20	68.52 (23.01)	68.76 (18.60)	0.96	83.44 (40.30)	91.26 (38.40)	0.51	0.55	9.10 (9.13)	8.64 (7.90)	8.59 (7.91)	7.62 (8.53)
Busselton (40)	744	31	64.47 (12.62)	65.58 (13.72)	0.63	66.73 (13.21)	65.13 (15.24)	0.51	0.54	−2.13 (2.20)	−2.33 (2.01)	−2.60 (2.34)	−2.82 (2.21)
CHS (35)	2,027	7	69.06 (17.08)	67.14 (11.25)	0.77	70.91 (17.27)	74.64 (17.51)	0.77	0.85	4.11 (6.41)	5.28 (4.18)	5.64 (4.43)	5.12 (5.36)
HUNT^*^ (37)	13,963	224	86.53 (23.56)	90.44 (18.90)	0.01	90.59 (21.21)	89.17 (22.86)	0.39	0.52	1.28 (1.31)	−0.53 (1.25)	−5.35 (1.67)	0.94 (1.51)
HealthABC (34)	1,881	26	73.02 (15.88)	77.03 (18.96)	0.20	84.36 (22.41)	80.99 (26.72)	0.68	0.83	−2.14 (4.43)	−6.94 (3.27)	−7.18 (3.27)	−4.33 (4.71)
InChianti^*^ (36)	790	85	79.98 (17.19)	84.20 (19.34)	0.03	75.14 (20.15)	74.74 (19.05)	0.57	0.56	1.78 (2.14)	−2.06 (1.88)	−4.78 (2.01)	−0.26 (1.95)
Leiden 85- study (32)	399	25	60.04 (13.77)	62.55 (17.06)	0.39	59.11 (15.27)	60.50 (16.31)	0.89	0.88	1.45 (3.15)	−1.10 (1.43)	−1.14 (1.43)	−1.40 (3.20)
PREVEND (41)	2,001	50	97.40 (14.92)	94.22 (14.95)	0.14	94.43 (15.06)	89.72 (13.92)	0.86	0.84	0.38 (1.73)	−1.02 (1.07)	−1.37 (1.12)	1.41 (1.39)
PROSPER^*^ (33)	4,822	180	57.60 (17.32)	53.22 (14.54)	0.00	58.44 (17.71)	54.51 (15.63)	0.92	0.95	0.37 (1.19)	0.47 (0.52)	0.48 (0.53)	3.76 (1.37)
AHS/RERF (42)	1,492	56	105.72 (25.80)	108.21 (23.58)	0.48	102.98 (26.38)	102.52 (27.17)	0.83	0.82	2.08 (3.37)	−2.07 (2.02)	−2.95 (2.10)	0.11 (3.30)
Rotterdam (43)	1,097	76	79.26 (15.73)	82.56 (19.47)	0.08	84.02 (27.97)	92.29 (31.71)	0.29	0.34	6.15 (2.13)	3.86 (1.85)	2.36 (1.97)	3.88 (2.24)
SHIP^*^ (44)	2,858	268	79.84 (14.25)	76.69 (15.09)	0.00	85.22 (21.29)	79.33 (20.30)	0.68	0.64	0.11 (1.18)	−0.74 (0.98)	−0.86 (0.97)	1.97 (1.17)

*Shyper, subclinical hyperthyroidism; ANCOVA, analysis of covariance; MD, mean difference; SD, standard deviation; SE, standard error*.

* *SHIP, PROSPER, InChianti, HUNT had p < 0.05 from the t-test, showing statistically significant baseline imbalance*.

Almost all studies had moderate correlation (≥50) between baseline and follow up outcome (Table 4). Results at study level for each statistical method are reported in Table 4 and those at meta-analytic level in Table 3. At study level, the studies that presented similar baseline outcome data between groups had small variation in the estimates throughout the methods. Among studies that showed imbalance baseline in the outcome between groups MDs varied more across methods. For example, in HUNT (37) the MDs were 1.28 for naïve, −0.53 for ANCOVA, −5.35 for change, and 0.94 for propensity score. We saw a similar pattern for InChianti (36), where MDs were 1.78 for naïve, −2.06 for ANCOVA, −4.78 for change, and −0.28 for propensity score (lower positive values indicate better renal function). Regardless of baseline imbalance, MDs for ANCOVA and change score always went to the same direction, while MDs from the propensity score approach varied.

For all studies, SEs were smaller for ANCOVA than other methods, indicating ANCOVA gave more precise estimates (Table 4). At the meta-analytic level, in the fixed effects model ANCOVA gave more precise pooled estimates (SE = 0.51) than other methods and the change score was less precise (with SE = 0.74), though no method identified an association between the renal function and subclinical hyperthyroidism (Table 3). In the random effects model, ANCOVA again showed more precise pooled estimates (SE = 0.53) and again the less precise was the change score (SE = 0.74). Heterogeneity was the highest when we used change score was used as outcome (τ^2^ = 3.18); it was lower for ANCOVA (τ^2^ = 1.00) and the propensity score (τ^2^ = 0.16) and it was null for the naïve approach. In both fixed and random effects, pooled results from propensity score showed less renal deterioration in the exposure group compared to the control group, while the other methods showed results in the other way round (Table 3 and Supplementary Figures 3, 4).

## Discussion

Among the published IPD-MA of NRSs in which continuous outcomes were assessed at baseline and follow-up (61% published since 2018), the change score was the most common statistical method, followed by ANCOVA—an unexpected finding because Cochrane recommends using ANCOVA to incorporate baseline outcome data in meta-analysis (45). A recent published paper by Tennant et al. also recommends not to use change score in studies that aim to estimate a causal-effect because their results are not meaningful unless the baseline exposure and baseline outcome are independent from each other, which is extremely unlikely in non-randomized studies (46). However, Tennant et al. also highlighted that adjustment for the baseline outcome, such as in ANCOVA, should not be made when the baseline outcome plausibly occurs after the exposure. In such cases, it would not generally be recommended to adjust for the baseline outcome, since such adjustment would not target the total causal effect of the exposure on the follow-up outcome and may introduce further bias. In other words, the adjustment strategy depends upon the causal scenario under consideration. We also compared the study and meta-analytic results from three statistical methods used to incorporate baseline outcome data in the analysis of a continuous outcome from two empirical examples of IPD-MA of NRSs. We considered ANCOVA, change score, propensity score. For comparison we also used the naïve approach that ignores the baseline outcome data. Study estimates varied across methods and depended on the balance/imbalance status of baseline outcome data between exposure and control group. When there was baseline imbalance, study estimates varied widely across methods, although estimates from ANCOVA and the change score flowed in the same direction. It is not necessarily expected that these two methods give results in the same direction, and we simply attribute that to the large sample size of the studies included in our examples (smallest study sample size was 229) that it is likely not to affect the sign of the point estimate. Studies with well-balanced baseline outcome data between groups had similar IPD MA results, regardless of the approach. We found ANCOVA gave the most precise estimates at both study and meta-analytic level, though at meta-analytic level the results for both examples did not differentiate across the methods. ANCOVA gave different results than propensity score adjustment: the propensity score seemed to overestimate the (positive) effect of the exposure group. One reason that may explain why the propensity score analysis does not generally agree with the ANCOVA analysis is the imbalance exposure “allocation ratio” that may produce a lack of overlap in the estimated propensity score by exposure groups and consequent extreme weights (47). Indeed, in our examples the proportion of participants in the euthyroid group is often much higher than those in the subclinical hyperthyroidism.

Overall, our findings are consistent with previous studies that suggested ANCOVA was most precise and better accounted for baseline imbalance between groups (1, 2). Our study adds further evidence in favor of using ANCOVA instead of change score when both baseline imbalance of the outcome data and correlation between baseline and change score are present (2, 5). Also in randomized studies where the exposure and baseline outcome variable are supposed to be unrelated, ANCOVA has been shown to be more efficient when compared with the change score, unless further adjustment for baseline outcome data is done in the change score approach (48). We extended on previous research comparing the propensity score approach to ANCOVA, the change score, and the naïve approach. We used IPD datasets from an international set of cohort studies with both small and large sample sizes so we could explore the effects of the methods in different scenarios.

Our study had three limitations. First, it did not assess the effect of the methods in both aggregate and IPD datasets. Second, for ANCOVA we assumed a linear confounding effect of baseline outcome data. However, association with follow-up may not be linear and a spline term may be included in the model to allow for potential non-linear confounding effect. Third, we only explored the effect of the methods in empirical examples; assessment via simulation studies may be further conducted.

For non-randomized studies that were well-balanced between groups, change score and ANCOVA performed similarly, but ANCOVA provided the most precise estimates at both study and meta-analytic level. In consistency with studies that showed biased estimates using change score in not randomized studies, we recommend using ANCOVA in meta-analyses of individual patient data from non-randomized studies.

## Data Availability Statement

The data analyzed follow restrictions of each included study cohort. For more information, see the link https://www.thyroid-studies.org/. Requests to access these datasets should be directed to Cinzia Del Giovane, cinzia.delgiovane@biham.unibe.ch.

## Ethics Statement

Ethical review and approval was not required for the study on human participants in accordance with the local legislation and institutional requirements. Written informed consent for participation was not required for this study in accordance with the national legislation and the institutional requirements.

## Author Contributions

LS, LW, and CDG have full access to all of the data in the study and take responsibility for the integrity of the data and the accuracy of the data analysis and had the final responsibility for the decision to submit for publication. LS, LW, CDG, and NR: concept and design. LS, LW, CM, and CDG: acquisition, analysis, or interpretation of data. LS and CDG: drafting of the manuscript and statistical analysis. CM, DB, AC, JG, WE, ST, RW, JJ, LF, GC, BÅ, LC, RP, MI, WO, BV, HV, JS, JW, RD, SB, MI, and NR: critical revision of the manuscript for important intellectual content. LS, LW, CM, DB, AC, JG, WE, ST, RW, JJ, LF, GC, BÅ, LC, RP, MI, WO, BV, HV, JS, JW, RD, SB, MI, and NR: administrative, technical, or material support. CDG: supervision. All authors have read and approved the final manuscript.

## Funding

The work from the Thyroid Studies Collaboration (TSC, www.thyroid-studies.org) was supported by grants from the Swiss National Science Foundation (SNSF 320030-172676 and 32003B_200606 both to NR). The Busselton Health Study had no financial support to disclose. The Cardiovascular Health Study (CHS) was supported by contracts HHSN268201200036C, HHSN268200800007C, HHSN268201800001C, N01HC55222, N01HC85079, N01HC85080, N01HC85081, N01HC85082, N01HC85083, N01HC85086, 75N92021D00006, and grants U01HL080295 and U01HL130114 from the National Heart, Lung, and Blood Institute (NHLBI), with additional contribution from the National Institute of Neurological Disorders and Stroke (NINDS). Additional support was provided by R01AG023629 from the National Institute on Aging (NIA). A full list of principal CHS investigators and institutions can be found at CHS-NHLBI.org. The European Prospective Investigation of Cancer (EPIC)-Norfolk study was supported by research grants from the Medical Research Council UK and Cancer Research UK. The Health, Aging and Body Composition (Health ABC) study was supported by NIA Contracts N01-AG-6-2101; N01-AG-6-2103; N01-AG-6-2106; NIA grant R01-AG028050 and NINR grant R01-NR012459. This research was funded in part by the Intramural Research Program at the NIA. The InChianti study was supported as a target project ICS 110.1jRS97.71 by the Italian Ministry of Health, and in part by the US NIA, contracts 263-MD-9164-13 and 263-MD-821336. The Trøndelag Health Study (HUNT) is a collaborative effort of HUNT Research Center (Faculty of Medicine and Health Sciences, NTNU, Norwegian University of Science and Technology), the Norwegian Institute of Public Health, Central Norway Regional Health Authority and the Trøndelag County Council. Thyroid function testing in the HUNT Study was financially supported by WallacOy (Turku, Finland). The Leiden 85-plus study was partly funded by an unrestricted grant from the Dutch Ministry of Health, Welfare and Sports (1997–2001). The original PROSPER study was supported by an unrestricted, investigator-initiated grant from Bristol-Myers Squibb. The Rotterdam Study was funded by the following: Erasmus MC and Erasmus University, Rotterdam, the Netherlands; the Netherlands Organisation for Scientific Research (NWO); the Netherlands Organisation for the Health Research and Development (ZonMw); the Research Institute for Diseases in the Elderly (RIDE); the Ministry of Education, Culture and Science; the Dutch Ministry for Health, Welfare and Sports; the European Commission (DG XII); and the Municipality of Rotterdam. The Radiation Effects Research Foundation (RERF), Hiroshima and Nagasaki, Japan, was a public interest foundation funded by the Japanese Ministry of Health, Labour and Welfare (MHLW) and the US Department of Energy (DOE). This publication was supported by RERF Research Protocol A5–13. SHIP was part of the Research Network of Community Medicine at the University Medicine Greifswald, Germany (www.communitymedicine.de), which was funded by the German Federal State of Mecklenburg–West Pomerania. The BELFRAIL study was funded by an unconditional grant from the Fondation Louvain. The Fondation Louvain was the support unit of the Université Catholique de Louvain in charge of developing education and research projects of the university by collecting gifts from corporate, foundations and alumni. The Brazilian thyroid study was supported by an unrestricted grant from São Paulo State Research Foundation (Fundacão de Amparo a Pesquisa do Estado de São Paulo) Grant 6/59737-9. The Prevention of Renal and Vascular End-Stage Disease (PREVEND) study has been made possible by grants from the Dutch Kidney Foundation: (E.033).

## Author Disclaimer

The content is solely the responsibility of the authors and does not necessarily represent the official views of the National Institutes of Health. The views of the authors do not necessarily reflect those of the two governments.

## Conflict of Interest

The authors declare that the research was conducted in the absence of any commercial or financial relationships that could be construed as a potential conflict of interest.

## Publisher's Note

All claims expressed in this article are solely those of the authors and do not necessarily represent those of their affiliated organizations, or those of the publisher, the editors and the reviewers. Any product that may be evaluated in this article, or claim that may be made by its manufacturer, is not guaranteed or endorsed by the publisher.
