# Supplementary material for: Incorporating Baseline Outcome Data in Individual Participant Data Meta-Analysis of Non-randomized Studies
Source: Front Psychiatry. 2022 Feb 22;13:774251. doi: 10.3389/fpsyt.2022.774251 (PMC8902696; doi:10.3389/fpsyt.2022.774251)
Supplement: Supplementary file 1 [file Data_Sheet_1.docx]

**Supplementary Material**

*Key words of the search strategy*:

“meta-analysis" or "Meta-Analysis" or "Meta analysis" or "meta analysis", "individual participant data" or "individual patient data" and exclude "randomized controlled trial" or "controlled clinical trial" or "randomized controlled trial" or "randomized controlled trials" or "controlled clinical trials" or "randomized controlled trials" or "Randomized controlled trial" or "Controlled clinical trial" or "Randomized controlled trial" or "Randomized controlled trials" or "Controlled clinical trials" or "Randomized controlled trials" or "randomized trial" or "randomized trials" or "Randomized trials" or "randomized clinical trials" or "Randomized Trials" or "randomised controlled trial" or "randomised controlled trial" or "randomised controlled trials" or "randomised controlled trials" or "Randomised controlled trial" or "Randomised controlled trial" or "Randomised controlled trials" or "Randomised controlled trials" or "randomised trial" or "randomised trials" or "Randomised trials" or "randomised clinical trials" or "Randomised Trials"

*Statistical methods to incorporate baseline outcome data in analysis of non-randomized studies*:

The models we applied are the following:

1. Analysis of covariance (ANCOVA): $Y_{ij}=\alpha_{ij}+\beta_{1i}x_{ij}+\beta_{2i}Y'_{ij}+e_{ij},$
2. Change score: $Y_{ij}-Y'_{ij}=\alpha_{ij}+\beta_{i}x_{ij}+e_{ij},$

Propensity score: We first calculated the predicted probability for each participant () of being exposed from the logit regression model *Pr* (*X*=1| $Y'$). Then we created the weights *W*_1_=1/ for the treated participants and *W*_2_=1/(1-) for the participants in the control group. Then we used the model of the naïve approach and weight the participants with the weights *W*_1_ previously derived.

For comparison we also applied the naïve approach: $Y_{ij}=\alpha_{ij}+\beta_{i}x_{ij}+e_{ij},$

Where *i* =1 to N depicts the number of cohorts and *j* shows the individual/patient participating of the respective cohort;

$Y_{ij}$represents the continuous outcome at follow-up;

$x_{ij}$ is the exposure variable that takes the value 0 when the individual/patient is in the control group and 1 when the patient belongs in the exposure group;

$Y'_{ij}$represents the continuous outcome at baseline.

The error term in all cases has the distribution of e ~ N(*0*, σ^2^).

*Figure 1. Forest plots for the association between subclinical hyperthyroidism and depressive symptoms from the fixed effect model*

**
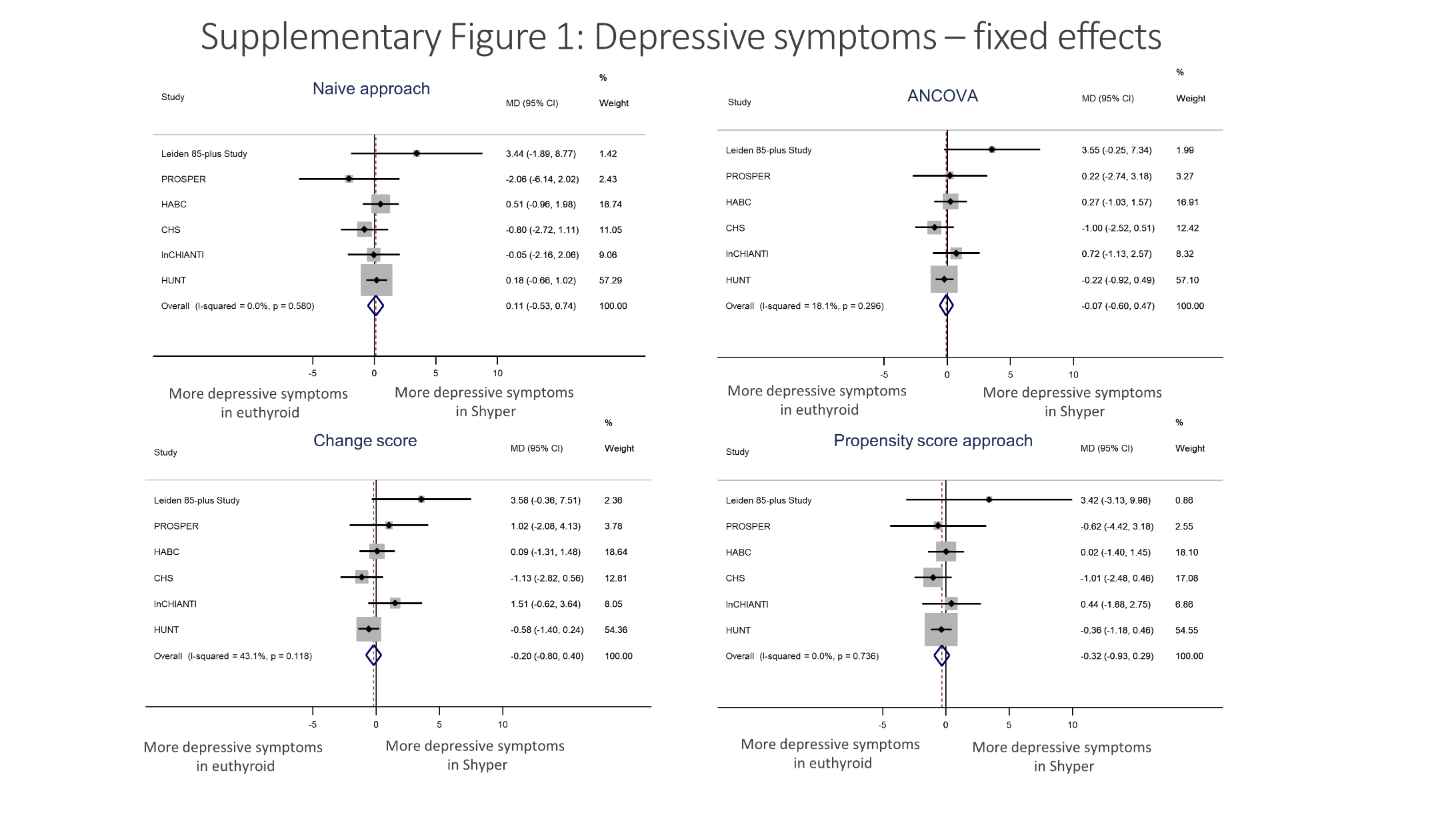
**

Shyper: subclinical hyperthyroidism; ANCOVA: Analysis of covariance; MD, mean difference; CI, confidence intervals.

*Figure 2. Forest plots for the association between subclinical hyperthyroidism and depressive symptoms from the random effects model*


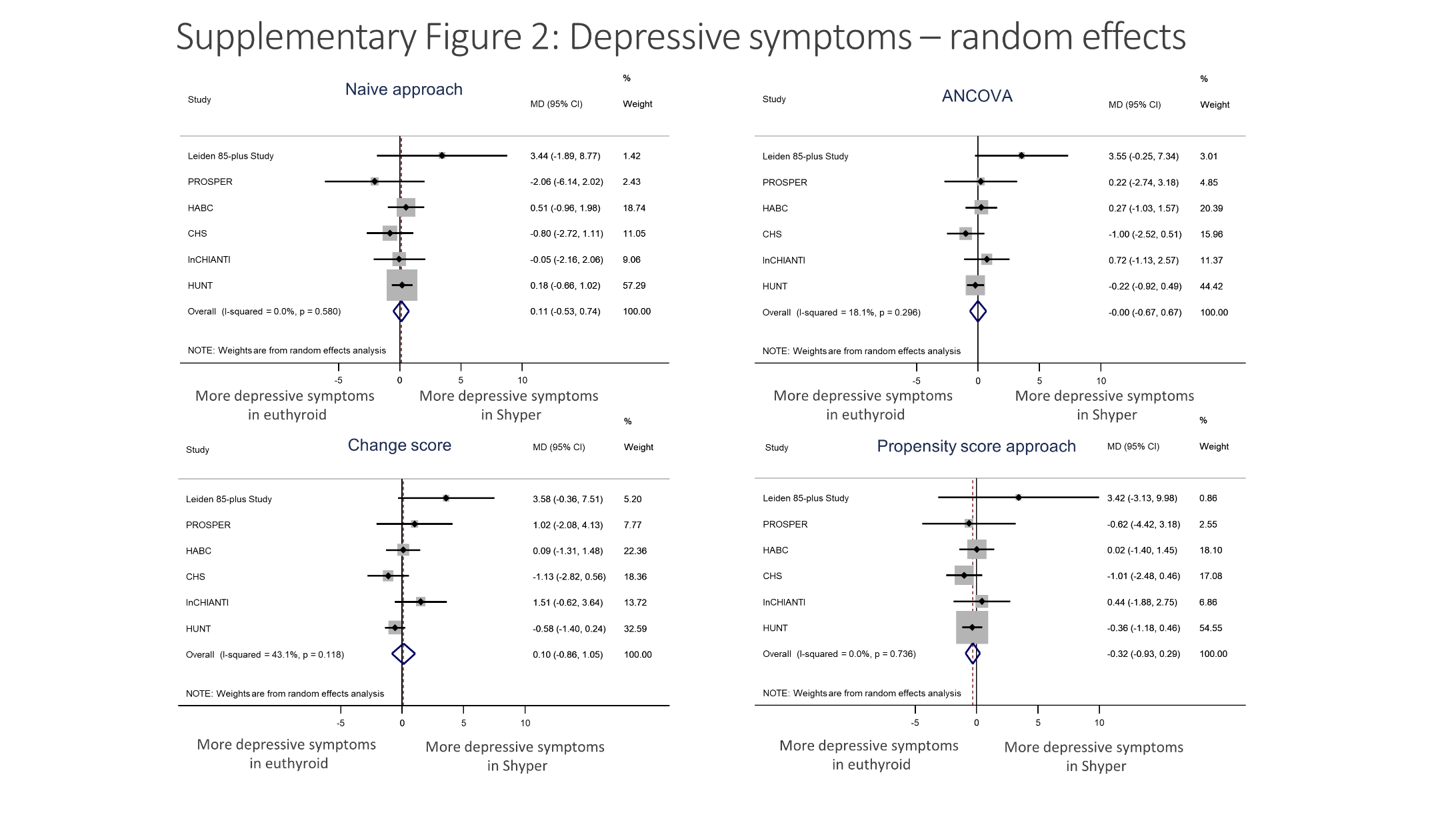


Shyper: subclinical hyperthyroidism; ANCOVA: Analysis of covariance; MD, mean difference; CI, confidence intervals.

*Figure 3. Forest plots for the association between subclinical hyperthyroidism and renal function from the fixed effect model*

**
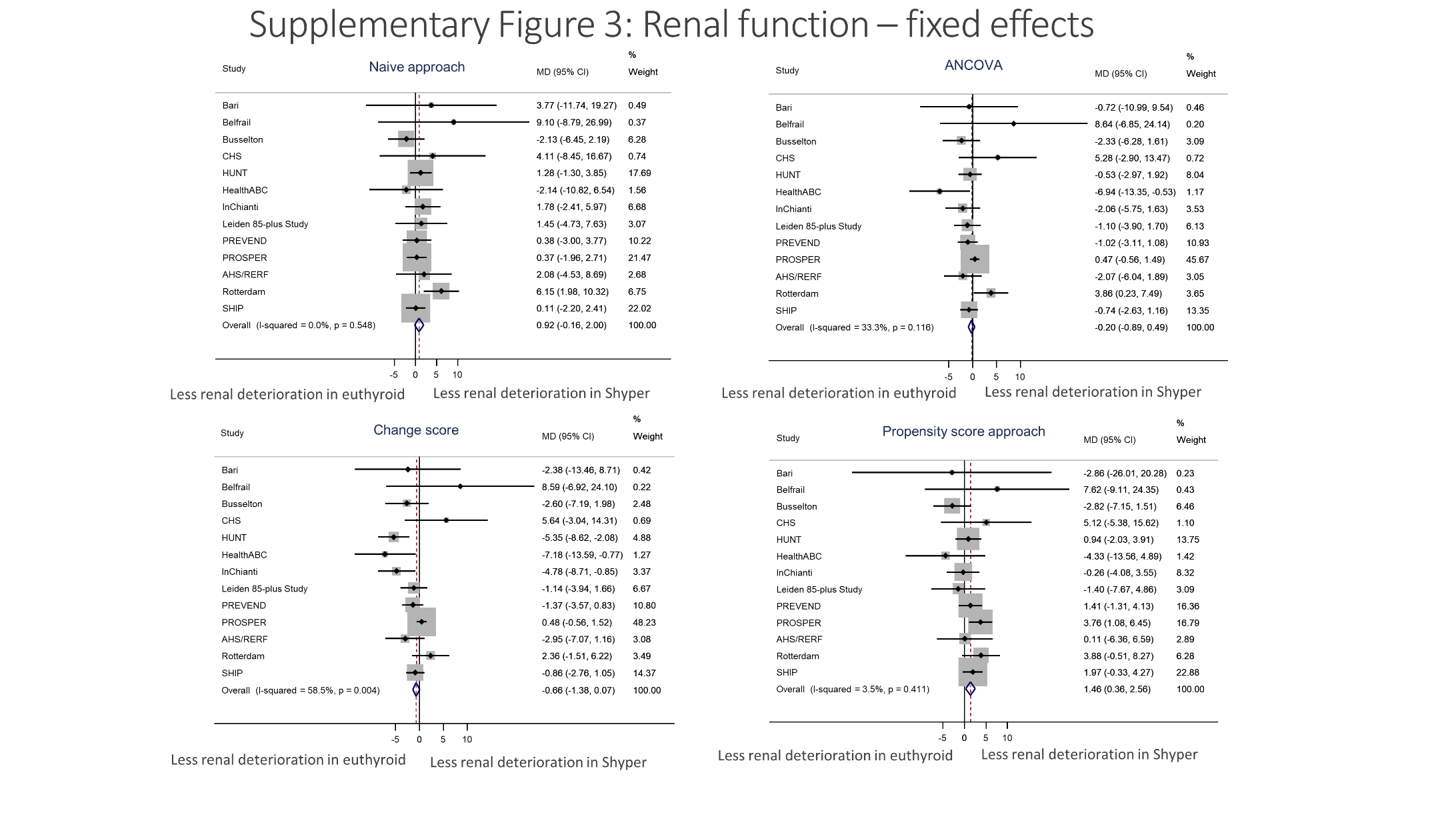
**

Shyper: subclinical hyperthyroidism; ANCOVA: Analysis of covariance; MD, mean difference; CI, confidence intervals.

*Figure 4. Forest plots for the association between subclinical hyperthyroidism and renal function from the random effects model*


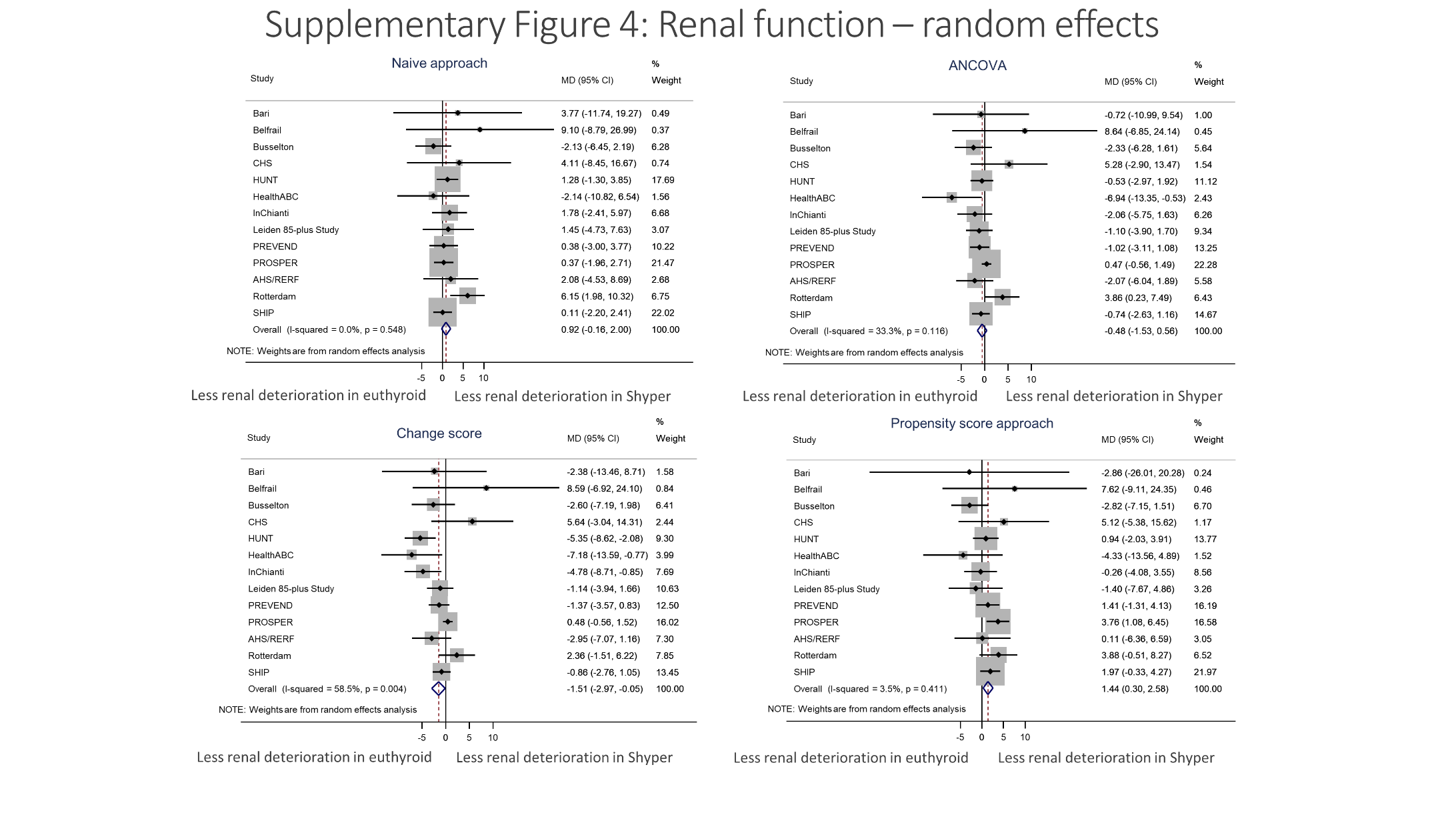


Shyper: subclinical hyperthyroidism; ANCOVA: Analysis of covariance; MD, mean difference; CI, confidence intervals.
